# Red blood cells are dynamic reservoirs of cytokines

**DOI:** 10.1038/s41598-018-21387-w

**Published:** 2018-02-15

**Authors:** Elisabeth Karsten, Edmond Breen, Benjamin R. Herbert

**Affiliations:** 1 Translational Regenerative Medicine Laboratory, Kolling Institute, Royal North Shore Hospital, Sydney, Australia; 2 Northern Clinical School, Faculty of Medicine, The University of Sydney, Sydney, Australia; 3 Sangui Bio Pty Ltd, Sydney, Australia; 4 Bioinformatic Consulting, Sydney, Australia

## Abstract

Red blood cells (RBCs) have been shown to affect immune function and can induce inflammatory responses after transfusion. The transfusion of washed RBCs can significantly reduce adverse effects, however, the soluble factors that may mediate these effects have not been identified. Previous studies have identified, but not quantified, a small number of chemokines associated with RBCs. We isolated RBCs from healthy volunteers and quantified of a panel of 48 cytokines, chemokines, and growth factors in the lysate, cytosol, and conditioned media of these cells using Luminex^®^ technology. This analysis revealed that, after correcting for white blood cell and platelet contamination, 46 cytokines were detected in RBC lysates, and the median concentration in RBCs was 12-fold higher than in the plasma. In addition, extensive washing of RBCs, such as that performed in proteomics analyses or prior to some RBC transfusions, significantly attenuated the release of six cytokines following incubation at 37 °C. This supports the hypothesis that, alongside its gas exchange function, RBCs play a role in cytokine signalling. This discovery may help supplement disease biomarker research and may shed light on adverse inflammatory processes that can follow RBC transfusion.

## Introduction

Analysis of clinical blood samples, specifically plasma or serum, is typically one of the first steps in diagnosing disease and is an important part of the research process of seeking new diagnostic markers. Testing only plasma or serum simplifies the analysis process by removing the cellular components of the blood. This is valuable, but it represents an incomplete profile of the relevant markers. The plasma is only one part of a much more complex, living tissue that rapidly responds to stress by altering the levels of signalling proteins that are also commonly studied markers^1^. After blood is collected for analysis, the sample must be stabilised to avoid changes in the plasma levels of disease markers. A number of factors, such as choice of anticoagulant, temperature, time, cell lysis, or platelet activation can result in the release of cytokines and other proteins into the plasma thus invalidating the resulting analysis^2^. An understanding and analysis of the other components of blood can provide some critical information in disease populations. Differential white blood cell counts and morphological changes in leukocytes can provide valuable diagnostic information that supplement other tests and clinical assessments^3^. These cells are known to produce and respond to a number of cytokines to either promote or suppress inflammatory processes^4,5^. Platelets are specialised, enucleate, cellular components of blood that function in the coagulation cascade. They are also an easily harvested source of cytokines and growth factors and have been harnessed as a therapeutic for inflammatory conditions^6^. Red blood cells (RBCs) on the other hand are understudied and are generally understood to be primarily involved in respiratory gas exchange. As a result of this and their extremely high haemoglobin content, which is considered a contaminant in biomarker studies, RBCs are discarded more often than they are analysed. However, some studies have now shown that RBCs are multifunctional and may be more complex than previously understood^7–9^.

In a recent study from our laboratory, it was determined that the pro-inflammatory cytokine and enzyme, macrophage migration inhibitory factor (MIF), was present in RBCs at levels 1000-fold higher than the typical plasma concentration^7^. As such, after blood collection, the plasma level of MIF could be significantly altered by relatively low levels of haemolysis. In addition, RBCs, or soluble factors released by these cells, can also stimulate the secretion of pro-inflammatory markers from lung fibroblasts^8^. These studies demonstrate that RBCs may be an important component of the immune system and are capable of signalling or receiving signal from other cell types. Neote *et al*. identified that the Duffy antigen receptor for chemokines (DARC) is “promiscuous” and has a number of binding targets^9^. These targets include some chemokines from the C-X-C or C-C cytokine families including IL-8, RANTES, and MCP-1. The authors proposed that the role of this receptor may be to act as a chemokine sink for modulating inflammation. Since this report in 1993, few additional cytokines and inflammatory proteins have been identified on or in RBCs^10–12^. Despite these studies, the role of RBCs in signalling remains poorly understood. In response, we investigated a panel of 48 cytokines, chemokines, and growth factors that are commonly studied as biomarkers of disease in the analysis of purified RBCs. Importantly, this analysis included identifying the total concentration in RBC lysates and the concentration released by intact RBCs and investigation into how the cytokine profile can be modulated.

## Results

### RBC purity following dextran sedimentation

Enriched RBCs were isolated from healthy volunteers using dextran sedimentation. Dextran sedimentation is a gentle technique that can be used to isolate leukoreduced RBCs^13,14^ and has been shown to produce a highly pure population as determined by flow cytometry^7^. The purity of the RBC population was calculated according to the level of white blood cell and platelet depletion from whole blood samples. Dextran sedimentation of whole blood resulted in 90.4 ± 11.0% and 98.7 ± 2.0% depletion of white blood cells and platelets respectively from the enriched RBC fractions.

### Cytokine profile of plasma and RBCs

RBCs have been identified in the literature as a reservoir for fewer than 10 cytokines^9–11^, but the levels have never been quantified. To identify if there were more cytokines associated with RBCs and to quantify the levels, the cytokine profile of RBCs, white blood cells, platelets, and plasma was determined using an antibody-mediated multiplex bead array. Of the 48 cytokines that were assayed for, 45 were present in the plasma fraction and 46 were present at detectable levels in the RBCs of one or more biological replicates after correction for white blood cell and platelet contamination (Tables 1–3). Across the 48 cytokines analysed, there were 42 where the level of the cytokine in RBCs substantially exceeded the plasma level (>4-fold increase). In these cytokines, the range of fold increase in RBCs versus plasma was 4.0 to 2445 (i.e. the RBC to plasma ratio) and the median fold change was 13.1.

**Table 1:** Concentration of pro-inflammatory cytokines in EDTA plasma and RBCs in one millilitre of whole blood with correction for white blood cell (WBC) and platelet contamination (*n* = 10), data are presented as mean ± SD.

Cytokine	Plasma^*^			RBCs^*^					WBC & platelet contribution		
	Subjects positive for cytokine	pg/mL of whole blood		Subjects positive for cytokine	pg/mL of whole blood		Corrected for WBC contamination (pg/mL)		pg/mL of total		Subjects positive for cytokine
		Concentration	SD		Concentration	SD	Concentration	SD	Concentration	SD	
Pro-inflammatory											
IFN-α2	10/10	10.9	*3.0*	6/10	546.6	*489.7*	544.8	*488.0*	4.5	*2.0*	4/10
IFN-γ	10/10	18.8	*9.7*	7/10	89.8	*54.2*	87.3	*55.3*	2.3	*2.7*	9/10
IL-1α	2/10	2.5	*0.3*	1/10	21.0	—	19.7	—	2.3	*1.7*	4/10
IL-1β	9/10	0.5	*0.2*	2/10	13.1	*4.2*	5.1	*5.7*	2.6	*4.7*	9/10
IL-5	8/10	2.4	*1.4*	3/10	28.2	*12.6*	28.2	*12.6*	—	—	0/10
IL-9	6/10	3.4	*2.4*	4/10	53.8	*42.1*	48.6	*41.0*	3.2	*4.1*	8/10
IL-12(p70)	8/10	2.6	*1.0*	3/10	19.8	*15.4*	15.0	*16.3*	3.9	*1.4*	6/10
IL-15	0/10	—	—	4/10	77.2	*54.8*	75.7	*54.8*	3.6	*2.4*	5/10
IL-17	6/10	6.3	*3.3*	7/10	91.5	*30.4*	78.6	*30.3*	17.5	*9.3*	10/10
IL-18	10/10	13.3	*10.8*	10/10	680.3	*318.4*	657.6	*322.2*	22.7	*29.2*	10/10
MIF	10/10	40.6	*46.5*	10/10	99,330	*47,473*	99,266	*47,491*	63.3	*59.7*	10/10
TNF-α	10/10	6.8	*3.1*	5/10	113.0	*79.2*	41.5	*56.7*	28.8	*36.5*	10/10
TNF-β	5/10	2.6	*0.9*	6/10	29.3	*8.0*	29.0	*7.9*	0.5	*0.3*	3/10
TRAIL	10/10	21.3	*10.7*	5/10	233.2	*85.5*	219.8	*84.8*	16.3	*9.8*	8/10

**Table 2:** Concentration of anti-inflammatory cytokines and chemokines in EDTA plasma and RBCs in one millilitre of whole blood with correction for white blood cell (WBC) and platelet contamination (*n* = 10), data are presented as mean ± SD.

Cytokine	Plasma			RBCs					WBC & platelet contribution		
	Subjects positive for cytokine	pg/mL of whole blood		Subjects positive for cytokine	pg/mL of whole blood		Corrected for WBC contamination (pg/mL)		pg/mL of total		Subjects positive for cytokine
		Concentration	SD		Concentration	SD	Concentration	SD	Concentration	SD	
Anti-inflammatory											
IL-1ra	9/10	17.1	*6.9*	4/10	732.6	*360.8*	463.3	*347.6*	1,048.4	*723.6*	10/10
sIL-2rα	10/10	26.8	*6.8*	7/10	257.9	*69.6*	254.7	*70.5*	5.2	*3.9*	8/10
IL-4	8/10	0.5	*0.4*	4/10	5.7	*1.6*	4.7	*2.0*	1.0	*0.6*	10/10
IL-10	2/10	2.0	*0.8*	0/10	—	—	—	—	0.5	—	1/10
IL-13	9/10	1.5	*1.0*	5/10	4.0	*2.9*	3.6	*3.2*	0.9	*0.1*	6/10
Chemokines											
CTACK	9/10	40.1	*15.3*	7/10	220.2	*73.1*	158.9	*76.7*	64.4	*50.7*	9/10
Eotaxin-1	9/10	9.5	*7.1*	10/10	108.1	*53.1*	104.7	*54.2*	3.4	*2.1*	10/10
GRO-α	7/10	17.9	*13.2*	8/10	359.7	*207.4*	335.1	*183.4*	35.1	*30.0*	7/10
IL-8	9/10	2.7	*2.0*	3/10	77.1	*60.1*	6.5	*18.3*	31.3	*44.6*	10/10
IL-16	10/10	59.9	*30.7*	9/10	5,082.9	*5,236.1*	5,080.7	*5,235.0*	6.6	*2.6*	3/10
MCP-1	1/10	5.6	—	6/10	160.0	*87.5*	150.1	*94.8*	11.5	*6.7*	10/10
MCP-3	8/10	16.4	*8.4*	4/10	86.4	*18.2*	85.6	*18.2*	1.7	0.2	2/10
MIG	10/10	299.5	*474.2*	4/10	1,235.4	*1,138.2*	1,231.0	*1,139.1*	5.7	*3.2*	5/10
MIP-1α	10/10	0.5	*0.3*	7/10	7.8	*5.6*	6.1	*4.3*	1.6	*1.8*	10/10
MIP-1β	10/10	6.2	*2.9*	6/10	64.0	*63.9*	56.9	*59.8*	7.4	*7.1*	10/10
RANTES	10/10	440.4	*258.8*	10/10	8,121.9	*9,905.4*	5,952.5	*8,982.4*	2,170.6	*1,699.9*	10/10
SDF-1α	10/10	468.5	*467.5*	10/10	3,086.3	*3,412.8*	3,038.1	*3,431.7*	60.2	*32.7*	8/10

**Table 3:** Concentration of growth factors and cytokines with multiple functions in EDTA plasma and RBCs in one millilitre of whole blood with correction for white blood cell (WBC) and platelet contamination (*n* = 10), data are presented as mean ± SD.

Cytokine	Plasma^*^				RBCs^*^				WBC & platelet contribution		
	No. of subjects	pg/mL of whole blood		No. of subjects	pg/mL of whole blood		Corrected for WBC contamination (pg/mL)		pg/mL of total		No. of subjects
		Concentration	SD		Concentration	SD	Concentration	SD	Concentration	SD	
Growth Factors											
bFGF	10/10	5.2	*2.5*	10/10	185.4	*58.6*	151.0	*68.1*	34.4	*24.6*	10/10
G-CSF	10/10	7.1	*4.6*	7/10	105.4	*75.2*	96.9	*70.9*	10.2	*7.8*	10/10
GM-CSF	0/10	—	—	9/10	936.4	*348.4*	825.8	*408.6*	127.1	*99.9*	10/10
HGF	10/10	66.0	*47.6*	10/10	938.9	*429.2*	831.1	*422.6*	107.8	*75.9*	10/10
IL-3	10/10	25.7	*10.5*	10/10	418.0	*182.6*	404.9	*186.4*	16.4	*8.2*	8/10
IL-7	9/10	2.6	*2.2*	6/10	22.3	*10.8*	21.9	*10.8*	0.6	*0.5*	5/10
IP-10	10/10	113.9	*111.2*	8/10	113.6	*83.0*	106.1	*78.2*	9.5	*7.6*	10/10
M-CSF	10/10	3.4	*2.7*	7/10	77.9	*36.0*	68.7	*30.3*	11.3	*7.7*	9/10
β-NGF	9/10	4.5	*4.1*	2/10	68.5	*6.8*	68.5	*6.8*	—	—	0/10
PDGF-bb	10/10	25.3	*15.5*	9/10	993.7	*1,525.3*	697.4	*1275.5*	729.1	*479.9*	10/10
SCF	10/10	41.6	*57.8*	3/10	125.8	*74.8*	125.1	*75.8*	1.9	*0.6*	4/10
SCGF-β	7/10	4,032.2	*4,773.8*	3/10	20,280.2	*9,245.3*	20,246.7	*9,298.2*	115.0	*60.5*	5/10
VEGF	8/10	2.8	*1.5*	10/10	110.7	*50.0*	84.2	*52.6*	26.5	*13.7*	10/10
Cytokines with multiple functions											
IL-2	0/10	—	—	2/10	13.7	*2.7*	13.7	*2.7*	15.9	*6.6*	6/10
IL-6	6/10	1.9	*1.4*	0/10	—	—	—	—	1.6	*0.7*	4/10
Il-12(p40)	3/10	33.2	*11.6*	7/10	1,603.7	*629.1*	1,475.8	*577.5*	203.9	*159.9*	10/10
LIF	8/10	5.5	*2.7*	3/10	119.5	*27.5*	119.5	*27.5*	—	—	0/10

The white blood cell/platelet fraction we analysed for the correction was comprised of both white blood cells and platelets at a ratio of 1:19 and was analysed in its entirety. Thus it is not known what level of cytokines came from either white blood cells or platelets alone. To correct for white blood cell and platelet contamination in the enriched RBC samples, the cytokine load of this fraction was determined and the levels were normalised according to the number of white blood cells in the enriched RBCs for each biological replicate (Tables 1–3). However, the ratio of contaminating white blood cells and platelets in these RBCs after dextran sedimentation was only 1:4 compared to 1:19 in the white blood cell/platelet fraction. Thus, the platelets were overrepresented by approximately 5× compared to the number actually contaminating the RBCs. This implies that the cytokine levels reported for the corrected RBCs is likely to be a conservative estimate of the actual concentration. This correction enabled an estimate of the RBC cytokine contribution to whole blood, wherein each replicate served as their own control. For some cytokines, the white blood cell and platelet contamination was negligible. For example, the total concentration of MIF in the RBC lysate was 99,330 ± 47,473 pg/mL, whilst the white blood cell/platelet contribution was 63.3 ± 59.7 pg/mL. This correction calculation had a more substantial effect on other cytokines. For example, the white blood cells and platelets contributed a third of the total CTACK measured in the RBC lysates (total concentration: 220.2 pg/mL; corrected concentration: 158.9 pg/mL).

In 1991, RBCs were reported to be a chemotactic sink for IL-8, and in 1993 the C-X-C class chemokines MGSA (aka GRO-α), NAP-2, and IL-8 were reported to bind to RBCs^9,15^. In the current study, of the 17 C-X-C chemokines in the family, five (29%) were identified in the lysates of RBCs after correcting for white blood cell contamination, specifically GRO-α, IL-18, IP-10, MIG, and SDF-1α (Table 4). Neote *et al*. also reported the presence of RANTES and MCP-1 of the C-C chemokine family^9^, and Fukuma *et al*. reported the presence of Eotaxin^10^. These findings were confirmed by the current study, which also identified RANTES, Eotaxin, and MCP-1 in RBC lysates. In addition, five more C-C chemokines were also identified out of a total of 27 chemokines in the family which adds up to 30% C-C chemokines being associated with RBCs (Table 4). This study has also revealed other classes of cytokines that are associated with RBCs as outlined in Table 4. In a recent study, RBCs were identified as a major source of the nuclear protein IL-33, which is part of the IL-1 cytokine superfamily^16^. They found that levels of IL-33 increased in the plasma of sickle cell patients who had experienced varying levels of haemolysis. The current study identified IL-1α, IL-1β, IL-1ra, and IL-18 in RBCs, which represents 36% of the IL-1 superfamily (Table 4). In addition to those already mentioned, a range of growth factors, chemokines, and pro- and anti-inflammatory cytokines were also identified (Tables 1–3).

**Table 4:** Families of cytokines that were identified in RBC lysates (*n* = the number of proteins in each family and *b*** = **the number of analytes available on the assayed Bio-Plex kits).

Family	Cytokine
C-C chemokines(*n* = 27; *b* = 7)	CTACK
	Eotaxin
	MCP-1
	MCP-3
	MIP-1α
	MIP-1β
	RANTES
CSF family (*n* = 3; *b* = 3)	G-CSF
	GM-CSF
	M-CSF
C-X-C chemokines (*n* = 17; *b* = 5)	GRO-α
	IL-8
	IP-10
	MIG
	SDF-1α
FGF growth factors (*n* = 22; *b* = 1)	bFGF
GM-CSF/IL-5/IL-3 family (*n* = 3; *b* = 3)	GM-CSF
	IL-3
	IL-5
IFN family (Type I) (*n* = 17; *b* = 1)	IFN-α2
IFN family (Type II) (*n* = 1; *b* = 1)	IFN-γ
IL-1 superfamily (*n* = 11; *b* = 4)	IL-1α
	IL-1β
	IL-1ra
	IL-18
IL-6 family (*n* = 11; *b* = 3)	G-CSF
	LIF
IL-12 family (*n* = 5; *b* = 2)	IL-12(p40)
	IL-12(p70)
IL-17 family (*n* = 6; *b* = 1)	IL-17
MIF family (*n* = 2; *b* = 1)	MIF
PDGF growth factors (*n* = 10; *b* = 2)	PDGF-bb
	VEGF
TNF superfamily (*n* = 19; *b* = 3)	TNF-α
	TNF-β
	TRAIL

**Figure 1:**
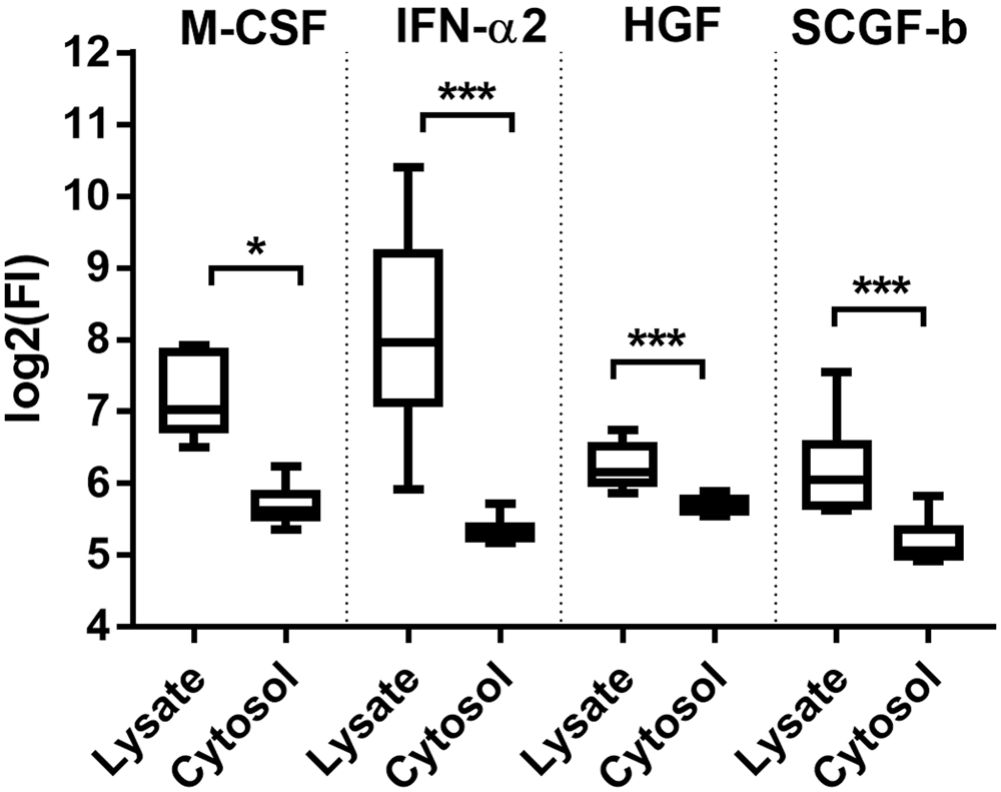
Cytokines in RBC lysate and cytosol. Level of cytokines in the total lysate or cytosolic fraction of RBCs as measured by Bio-Plex and reported as fluorescence. Data are presented as mean with minimum and maximum values (*n* = 10). Data are significant if *p* < 0.05 (*), *p* < 0.01 (**), or *p* < 0.001 (***).

A small number of chemokines are known to bind to and localise on the RBC membrane through ligation of DARC^9,15^, however a detailed investigation in the literature of how the remaining cytokines are partitioned in RBCs is yet to be conducted. We sought to identify which, if any, of the cytokines were associated with the membrane fraction of RBCs. To achieve this, the cytokine profile of the cell lysates (containing the cell membranes) was compared to the cytokine profile of the cytosolic fraction (lacking the cell membranes). There was a significant lower amount of four cytokines in the cytosolic fraction (Fig. 1), suggesting that these cytokines were, at least in part, associated with the cell membrane. These cytokines included HGF, IFN-α2, M-CSF, and SCGF-β.

### RBC cytokine binding

**Figure 2:**
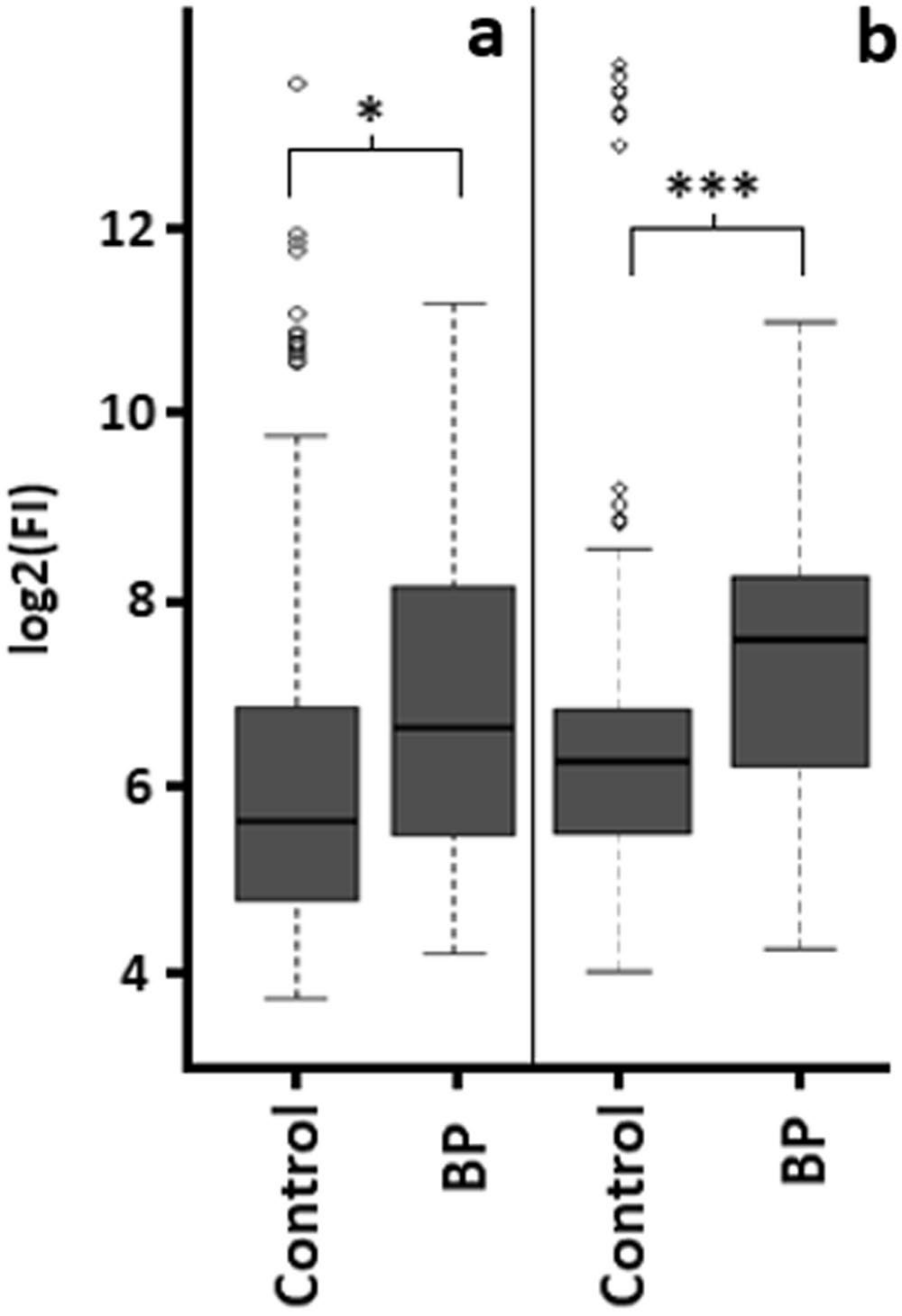
Incubation of recombinant cytokines with RBCs. Boxplot summaries of cytokine log2 transformed raw fluorescence response data for RBC lysates collected using (**a**) the Bio-Plex human 27-plex panel and (**b**) the Bio-Plex human 21-plex panel (*n* = 5). Cells were incubated for 24 hours (37 °C) with or without recombinant cytokines (BP, and control, respectively). Data are statistically significantly different if *p* < 0.05 (*), or *p* < 0.001 (***).

It was of interest in this study to investigate if RBCs were able to bind these cytokines. RBCs were incubated for 24 hours (37 °C) in the presence of a recombinant cytokine standard mix (Bio-Plex kits, Bio-Rad). As indicated in Fig. 2, RBCs that were incubated with recombinant cytokines had significantly higher cytokine concentrations in the resulting lysates across both panels of 48 cytokines when compared to controls (*p* = 0.038 for 27-plex, and *p* = 8.6 × 10^9^ for 21-plex). RBCs have been observed to sequester IL-8 out of whole blood, and subsequently inactivate the chemokine, until cellular saturation through ligation of DARC^15^. Similar reports have been made for MCP-1 and MIP-1α^17,18^. Our data support these findings and suggest that the RBCs sequestered at least a portion of the recombinant cytokines out of solution.

### Recovery efficiency of RBC lysates

To determine if assaying the RBC lysates could have been confounded by sample interference, the recovery efficiency of RBC lysates was determined by spiking in known concentrations of recombinant cytokines and measuring the final yield. A result of <100% indicated that the assay was not detecting all of the available cytokines, and a recovery efficiency of >100% indicated that the assay was reporting more cytokine than was actually present. For this study, the mean recovery efficiency in RBC lysates for 39 of 48 of the cytokines fell between 70 and 120% and approximately 66% of these cytokines had mean recovery efficiencies between 90 and 110% (Supplementary Table S1-S2). There were two cytokines that had mean recovery efficiency of over 120%, IFN-γ (122.9 ± 6.2%) and IL-12(p40) (140.5 ± 41.5%). Quantitative data collected for these two cytokines should be used with caution. The recovery efficiencies reported here are similar to reported values for plasma assayed on the Luminex platform^19,20^. Belabani *et al*. reported on the accuracy of Luminex based multiplex assays and their acceptance criteria for recovery efficiencies was determined to be between 75–125%^19^. Using that criteria, 38 of the 48 cytokines analysed in this study fell within the acceptable range for RBC lysates.

### Cytokine profile of RBC conditioned media

**Figure 3:**
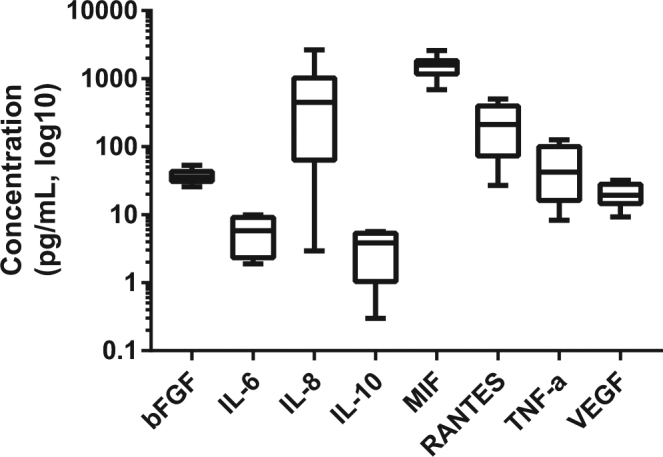
Cytokines in RBC conditioned media. Concentration of cytokines in the conditioned media of RBCs (400 × 10^8^ cells per mL) incubated in PBS for 24 hours at 37 °C as measured by Bio-Plex. Data are presented as mean with minimum and maximum values (*n* = 10).

Following the identification of a variety of cytokines in RBC lysates, it was of interest to determine if RBCs would also release these molecules if incubated in a protein free media (PBS) for 24 hours. The resulting conditioned media was assayed using Luminex® technology. The number of RBCs used to produce the conditioned media was approximately 25 × lower than the concentration of cells in whole blood (whole blood contains approximately 5 × 10^9^ RBCs/mL). Even so, detectable levels of 46 of the 48 cytokines were present in the conditioned media for one of more biological replicates (Fig. 3). The aim of this experiment was to identify and quantify which cytokines would be released from RBCs in the absence of anything that could stimulate a negative feedback loop, such as the presence of plasma. Plasma contains a variety of cytokines, and differing levels of these are likely to impede the release of cytokines. In addition, to ensure the RBCs were not lysing during this time, haemoglobin levels were monitored and determined to be minimal as outlined below. The detectable concentration of IL-8 varied substantially between biological replicates; the range of IL-8 in the conditioned media was 2.9–2641 pg/mL. This variability was also observed for a number of other cytokines including RANTES (27–502 pg/mL), TNF-α (8.3–127 pg/mL), and MIF (688–2598 pg/mL). For IL-10 and IL-6, levels were undetectable in RBC lysates, but were present in the RBC conditioned media (3.4 ± 2.3 pg/mL for IL-10 and 5.9 ± 3.4 pg/mL for IL-6). The cytokines that exhibit this increase in concentration may have been bound to chaperones or to a complex when the lysate was analysed but were released when the cells were incubated over 24 hours. The limitations of immunoassays in detecting complex bound cytokines have been previously described^21^. Furthermore, there is evidence that some molecules are contained within RBCs in an inactive form so that they are ready for rapid release^22^. Oonishi *et al*. identified that only after mechanical stress, were PGE_1_ and PGE_2_ detectable in RBC cytosols^22^. They theorised that the molecules were not being manufactured, but instead that enzymes were converting the molecules into a form that then rendered them active and detectable. The concentrations of released cytokines are summarised in the Supplementary results (Supplementary Table S3–S4). Analysis of RBC conditioned media has not been widely reported in the scientific literature, but its effect on T cells and fibroblasts has been briefly reported^8,23^. However, the cytokine profile of the conditioned media in these studies was not investigated. We hypothesised that the cytokine profile of the RBC conditioned media could be manipulated by excessive sample processing, we thus tested the effect of multiple wash cycles on the release of cytokines from intact RBCs.

### Cytokine profile of washed RBCs

Amongst the proteomic analyses of RBCs, no studies have yet identified the presence of cytokines as reported in this study. There are likely to be a number of reasons for this such as issues with the dynamic range of the proteins; the abundance of these cytokines is approximately 100 million-fold lower than the abundance of haemoglobin in RBCs. In addition, the detection of low abundance proteins will be hindered by the vigorous washing procedures typically done during proteomic sample preparation. As an example, in a report on the analysis of the proteome of RBC membranes, the isolated cells were centrifuged, filtered, washed four times in an isotonic buffer, and finally the cells were lysed and the resulting membranes were washed another four or five times^24^. This processing method may be optimal for the identification of transmembrane or structural proteins, but in the identification of cytokines bound to the surface of the cell membrane this is unlikely to be effective.

**Figure 4:**
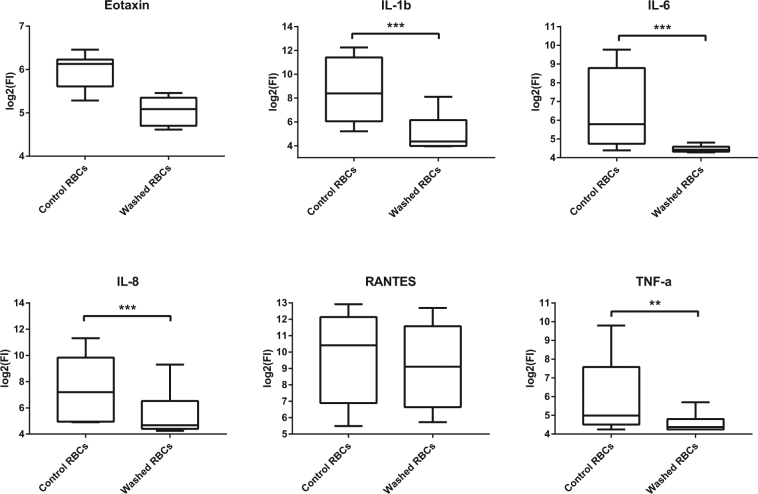
Cytokines in cell lysates and conditioned media following cell washing. Concentration of cytokines in the conditioned media of RBCs washed once (control RBCs) or ten times (washed RBCs) and subsequently incubated in PBS (400 × 10^6^ cells per mL) for 24 hours at 37 °C as measured by Bio-Plex. Data are presented as mean with minimum and maximum values (*n* = 10). Data are significant if *p* < 0.05 (*), *p* < 0.01 (**), or *p* < 0.001 (***).

To study the effect of aggressive sample handling, we washed intact RBCs ten times in an isotonic buffer, which significantly attenuated the release of six cytokines into the RBC conditioned media (Fig. 4) including IL-1β, IL-6, IL-8, MIP-1α, MIP-1β, and TNF-α. Notably, these results were observed without a significant change in RBC purity (94.7 ± 4.8 and 96.3 ± 4.2 white blood cell depletion for one wash and ten washes respectively, p = 0.24).Changes in the cytokine profile following extensive processing has similarly been observed for platelets wherein, excessive washing or vigorous processing techniques have been shown to promote their degranulation and subsequently an increase in the secreted detectable cytokine levels^25^. Although the platelet literature is clear on the effects of processing, the only other enucleate cells in humans, RBCs, are regarded as transcriptionally and translationally inert and incapable of secretory activity.

### Haemoglobin quantification

To determine if the cytokines that were detected in the conditioned media were a result of cellular release or simply haemolysis, the concentration of haemoglobin in RBC conditioned media was quantified by assessing the absorbance of plasma and RBC conditioned media samples at 414 nm. The level of haemoglobin in the conditioned media samples was significantly lower (p < 0.0001) than the haemoglobin concentration of the plasma samples (0.16 ± 0.09 and 0.54 ± 0.24 mg/mL respectively). The mean concentration of haemoglobin in the conditioned media samples corresponded to approximately 1.4% of the original RBC number, which is too low to explain the concentration of 43/46 cytokines detected in the conditioned media (Supplementary Table S1).

## Discussion

Unlike any other study to date, the concentrations of cytokines in RBC lysates have been quantified and reported according to their relative abundance in whole blood (Tables 1–3). The results of this study revealed that 46 of the 48 cytokines assayed were detected in RBC lysates of healthy volunteers (Tables 1–3) and for 42 of these cytokines, they were more than 4-fold higher in the RBC lysates than in the plasma with a median fold increase of 13.1 (range 4.0 to 2445-fold). This panel of cytokines identified in RBCs cover a range of pro- and anti-inflammatory factors, chemokines, and growth factors. A number of these cytokines are regularly monitored in plasma or serum for biomarker studies^1^ and the expression and secretion of these cytokines are thought to be limited to specific subsets of white blood cells^26^. The identification of these cytokines associated with RBCs indicates that an additional, previously unknown, level of cytokine interactions may exist in blood. In clinical studies, optimal sample preparation is crucial for reproducible results^27^. To achieve this, techniques generally focus on minimising white blood cell and platelet activation during plasma or serum isolation. However, the results of this study highlight that even minimal haemolysis that may not be macroscopically detectable (haemolysis of ≥0.125% of RBCs in whole blood^28^) could interfere with the results of the clinical study. For example, IL-16 was present at 85 times the concentration in RBCs than in plasma. Haemolysis of 0.125% of RBCs (releasing approximately 0.19 mg/mL of haemoglobin) could release approximately 6 pg/mL of IL-16, which is 10% of the mean plasma concentration (59.9 pg/mL). Thus, an undetectable amount of haemolysis would quickly confound results of a single assay and introduce inter-sample variability.

After incubation, a wide range of cytokines were detected in the conditioned media of RBCs (Fig. 3). The concentration of haemoglobin in the RBC conditioned media indicated lysis of 1.4% of the original number of RBCs. For 43 cytokines, the concentration in the conditioned media was higher than what could be attributed to the RBC lysis. This suggests that the proteins were being released or shed in a mechanism that is independent of haemolysis. Very few studies have determined the content or activity of RBC conditioned media. One study reported that T cell proliferation was stimulated in the presence of RBC conditioned media and that this stimulation was comparable with intact RBC-mediated proliferation^23^ and another observed that culturing fibroblasts in the presence of intact RBCs or RBC conditioned media stimulated the secretion of IL-8 and the expression of the corresponding mRNA^8^. This result was also replicated by culturing fibroblasts in the presence of TNF-α and IL-1β, both of which were cytokines detected in the RBC conditioned media in this current study (Fig. 3 and Table S3–S4). As is typical of the literature on RBCs, the cytokine profile of the conditioned media was not investigated, let alone quantified, in any of these studies. When normalised for cell number, the concentration of 28 of the cytokines in the conditioned media were higher than that detected in the RBC lysates prior to incubation. The cytokine with the largest difference between pre-culture lysate concentrations and the concentration in the conditioned media was IL-8 (Tables 1–3 and Fig. 3). With white blood cell correction, IL-8 was almost undetectable in the RBCs lysates prior to incubation (6.5 ± 18.3 pg/mL). After incubation at 37 °C for 24 hours, 657.1 ± 788.2 pg/mL of IL-8 was released in the RBC conditioned media. The lack of detectable IL-8 in the RBC lysates was unexpected as it is one of the few cytokines that has been identified by multiple reports as being present in and released from RBCs^15,23,29^. An increase in secreted cytokines from stored RBCs is generally attributed to white blood cell contamination, but a 1999 study suggested otherwise^29^. That study compared the cytokine release after storing whole blood for 42 days to RBCs and leukodepleted RBCs^29^ and they reported that the storage of whole blood resulted in the lowest concentration of secreted IL-8 (1.7 ± 7.0 pg/mL) and that the leukodepleted RBCs secreted the highest concentration of IL-8 (26.8 ± 31.0 pg/mL). Further to that, warming the leukodepleted RBCs to room temperature for 24 hours during the 42-day period resulted in even higher IL-8 release (125.8 ± 158.6 pg/mL). These results suggest that perturbation of these cells stimulated the release of IL-8. In light of this study, it is likely that the reason for the increased cytokine concentration in the stored RBC conditioned media is controlled release by intact RBCs. Free IL-8 has a serum half-life of minutes, however, studies have shown that there is a dynamic equilibrium between monomers, dimers, and cell-surface bound forms of IL-8^30^. Although, in principle, RBC lysis releases the cytosolic contents for analysis, proteins such as IL-8 may be released from lysed RBCs in a bound form that renders them inaccessible to immunoassay. Using PBS for the production of the conditioned media is a limitation of this study, as it is not directly relevant to what will be occurring *in vivo*. Future studies in understanding the dynamic processing occurring between plasma and RBCs would be of interest and could be achieved using labelled cytokines or cells.

This study revealed that washed RBCs have significantly attenuated release of a variety of cytokines (Fig. 4). Notably, washing RBCs appears to have some therapeutic benefit in transfusion medicine. The Australian Red Cross Service offers washed RBCs for specific indications including patients with allergic reactions or in response to severe reactions with unwashed RBC packs. In fact, washing RBCs following storage appears to be correlated with reduced incidence of adverse events and in some cases, improved outcomes such as in mice with haemorrhagic shock^31^. One of the reported benefits of this procedure is the depletion of inflammatory proteins from the blood pack^31^. This depletion is likely achieved by both removal of the storage media which contains inflammatory proteins^31^, and also by the removal of loosely bound cytokines from the surface of RBCs, as observed in this study (Fig. 4). Cholette *et al*. reported that children undergoing cardiac surgery had reduced post-operative inflammation following transfusion of washed RBCs compared to transfusion of unwashed cells^32^. This effect was represented by resulting lower serum levels of inflammatory cytokines and a change in trend towards reduced mortality^32^. However, there appears to be a delicate balance required in the amount of washing needed to achieve this therapeutic benefit. Excessive washing increases RBC osmotic fragility and can lead to increased haemolysis following transfusion which can cause downstream adverse effects^33–35^.

The results of this study may have implications on understanding the pathology of haemolytic conditions such as sickle-cell disease. In these disorders, the increase in free haemoglobin in the plasma has been attributed to causing complications such as platelet activation and inflammation^36,37^. In light of our results, haemolysis would also lead to the release of a range of cytokines into the plasma that may have down-stream effects on neighbouring cells. These results also suggest that RBCs may play a crucial role in blood in regulating circulating cytokine levels by releasing signalling proteins and, coupled with the capacity to also bind these proteins through DARC^9,15^, the cells may be contributing to blood homeostasis. If RBCs are indeed contributing to blood homeostasis through binding and release of cytokines, the mechanism behind this process is currently unknown. RBCs are devoid of the classic secretion pathways that are well documented for other cell types due to the lack of organelles. However, other mechanisms have been suggested in the literature that may have a role in this process including coagulation, protease activity, and release of microparticles^15,17,22,38^. Studies of platelets indicate that differences in manual handling and the presence of factors such as calcium or thrombin affect the cytokine release^39^. Platelet studies may provide a useful guide when considering further experiments on RBC cytokine binding and release. Considering the number of cytokines in different families identified in this study (Table 4), it is unlikely that a single mechanism will apply to all of them.

If RBCs contain circulating plasma proteins, then they may too be representative of the inflammatory state of the participant which has potential implications in diagnostics. Small volume blood collection is a crucial step in the development of rapid, bench side diagnostics, and the use of RBCs for diagnostic purposes may be the platform to enable this due to their relatively high abundance. With the increasing use of immunoassays, the need to standardise methods for sample processing has become apparent. Many of the techniques currently in use are designed to prevent potential confounders such as platelet activation or protease degradation. This study has highlighted the possibility that the release of cytokines from RBCs, either by haemolysis or from intact RBCs, may be another potential confounder that needs to be addressed. This may be achieved by preventing haemolysis, monitoring levels of haemoglobin in the plasma or serum, or else by reducing the sample processing time and the number of handling steps to minimise the release of cytokines from intact RBCs. This study may be based on experiments on isolated blood components, but they make one thing quite clear. The locations and total quantities of signalling molecules in blood such as cytokines are different to those described in the literature and as such, the signalling processes in blood are probably more complex than currently understood. In one aspect, the conclusion from these experiments is that a reductionist biomarker discovery approach of analysing plasma or serum in isolation may be limited, and a large piece of the puzzle may be lost in the process. Techniques such as monitoring the relative levels of panels of cytokines *between* the cell types and plasma in blood may prove to be more beneficial in disease research as opposed to determining the absolute concentrations in plasma or serum alone. Blood and its signalling network is highly complex, and analysis of individual cytokines is unlikely to be representative of the whole picture.

## Methods

### Blood donation

This study was approved by the Macquarie University Human Research Ethics Committee (5201100827) and by the Northern Sydney Coast Human Research Ethics Committee of NSLHD and CCLHD (1201–046 M) and was carried out in accordance with the relevant guidelines and regulations. Written informed consent was collected from all participants before participation in this study. Whole blood was collected from healthy volunteers by venepuncture (*n* = 42; female: 20, male: 22) directly into K_2_EDTA vacutainers (BD Biosciences, San Jose, CA). The age range of these participants was 20–63 years. All fractions of blood were collected and processed at room temperature within one hour of collection.

### Plasma and blood cell isolation

Plasma and blood cells were isolated from EDTA anti-coagulated whole blood. Plasma and white blood cells at known concentrations were collected and frozen immediately for future analysis. RBCs were collected and were either frozen immediately or were used fresh for conditioned media and washing experiments at known concentrations. Cell number and purity was determined using a haematology analyser (Coulter AcT Diff, Beckman Coulter, Brea, CA). Plasma was isolated by centrifugation of whole blood (1500 *g*, 10 minutes) and the plasma fraction was collected and stored at −80 °C. Red and white blood cells, and platelets were isolated using dextran sedimentation as follows. Whole blood was added to a dextran solution (450–650 kDa, 6% in 0.15 M sodium chloride) at a 2:1 ratio (blood:dextran). This cell suspension was left upright at room temperature for 1 hour until the RBCs sedimented to the bottom of the tube after which the upper white blood cell rich layer and the lower RBC fraction were collected into individual tubes. The RBC fraction was washed once in phosphate buffered saline (PBS, 500 *g*, 5 minutes) and an aliquot of these RBCs were then washed nine more times in PBS by inversion and centrifugation at 1500 *g* for 5 minutes without a brake. RBCs were either frozen in PBS at −80 °C to produce cell lysates or else were used fresh for conditioned media experiments. The cytosolic fraction was isolated from RBC lysates by removing the cellular membranes (16,000 *g*, 20 minutes). For isolation of the white blood cells and platelets, the upper white blood cell rich layer following dextran sedimentation was washed once in PBS (1000 *g*, 10 minutes). The supernatant was discarded, and any contaminating RBCs were eliminated by hypotonic lysis with 0.5 mL of Milli-Q water for 30 seconds. After this time, isotonicity was restored by adding 1 mL potassium chloride (0.65 M) and the cell suspension was diluted up to 15 mL with PBS. The remaining cells, which comprised of both white blood cells and platelets, were then washed twice in PBS (1000 *g*, 5 minutes) before use. Isolated white blood cells were frozen in PBS at −80 °C to produce lysates.

### Binding of recombinant cytokines to RBCs

Bio-Plex recombinant cytokine standards (27-plex and 21-plex human cytokine panels, Bio-Rad, Hercules, CA) contain cytokines at variable concentrations so these were reconstituted and diluted in PBS according to manufacturers instructions to produce standard-3 (27-plex and 21-plex human cytokine panels, Bio-Rad). Bio-Plex standard-3 was then diluted 1:8 in PBS with RBCs at a final concentration of 400 × 10^6^ cells/mL and the cell suspension was incubated for 24 hours (37 °C). Following incubation, the RBCs were isolated (500 *g*, 5 minutes), frozen at −80 °C in PBS, and were subjected to three freeze-thaw cycles to ensure complete cellular lysis.

### Recovery efficiency

The recovery efficiency of the Bio-Plex recombinant cytokines standards (Bio-Plex Pro 27-plex, and 21-plex cytokine panels, Bio-Rad, Hercules, CA) in RBC lysates was determined. The cytokine standards were reconstituted and diluted in PBS according to manufacturers instructions to produce standard 3 (Bio-Plex Pro 21-plex and 27-plex cytokine panels). Subsequently, 25 µL of the diluted recombinant cytokine standards or PBS alone were spiked into 75 µL of the lysates (diluted in PBS to 400 × 10^6^ cells/mL). These samples were analysed using the 27-plex and 21-plex human cytokine panels (Bio-Plex Pro, Bio-Rad) as outlined below. The recovery efficiency for each cytokine was calculated using Equation (1).

Recombinant protein recovery efficiency (%).

1 $$\frac{[Analyte]\,in\,control\,lysates+[rAnalyte]}{[Analyte]\,in\,spiked\,lysate}\times 100$$

### Red blood conditioned media

After isolation, RBCs were incubated for 24 hours in PBS (400 × 10^6^ cells/mL, 37 °C, 5% CO_2_). After incubation, the conditioned media was collected by centrifugation (500 *g*, 5 minutes) and all samples were stored at −80 °C and were subjected to three freeze/thaw cycles before analysis.

### Cytokine and haemoglobin quantification

All samples were stored at −80 °C and were subjected to three freeze-thaw cycles to ensure complete cellular lysis prior to analysis. Two multiplex assays were used in this study, the first was the 27-plex human cytokine panel that assays for FGF basic, Eotaxin-1, G-CSF, GM-CSF, IFN-γ, IL-1β, IL-1ra, IL-2, IL-4, IL-5, IL-6, IL-7, IL-8, IL-9, IL-10, IL-12(p70), IL-13, IL-15, IL-17, IP-10, MCP-1, MIP-1α, MIP-1β, PDGF-BB, RANTES, TNF-α, and VEGF (Bio-Plex Pro 27-plex, Bio-Rad), and the second was the 21-plex human cytokine panel that assays for IL-1α, sIL-2Rα, IL-3, IL-12(p40), IL-16, IL-18, CTACK, GRO-α, HGF, IFN-α2, LIF, MCP-3, M-CSF, MIF, MIG, β-NGF, SCF, SCGF-β, SDF-1α, TNF-β, TRAIL (Bio-Plex Pro 21-plex, Bio-Rad). The assays were performed according to manufacturer’s instructions using an automated magnetic wash station (Bio-Plex Pro II, Bio-Rad) for the washing steps. The assays were run on the Luminex® 200^TM^ system (Bio-Rad) and fluorescence values were collected. The calibration curve for each cytokine was analysed with 5 parametric logistic curve regression using Bio-Plex manager software (ver. 5.0, Bio-Rad). Standard values were considered acceptable if the points fell within 80–120% of the expected values. The concentration of free haemoglobin in plasma samples and RBC conditioned media samples were monitored by assessing absorbance at 414 nm (Synergy 2 plate reader, BioTek, Winooski, VT) as previously described^24^ with a calibration curve of purified haemoglobin (Sigma Aldrich, St Louis, MO). RBC membranes were removed from the conditioned media samples prior to analysis by centrifugation (16,000 *g*, 15 minutes) and the resulting data were analysed on GraphPad Prism software (ver. 6, USA).

### Statistical analysis

The multiplex assay data are presented as either concentration or fold change of fluorescence. Haemoglobin concentration of plasma and conditioned media samples were statistically evaluated using two-tailed, paired t-test to assess statistical significance (*p* < 0.05) between groups. Graphing of results was performed using GraphPad Prism software (ver. 6, USA). The concentration of cytokines in blood components was presented according to the relative contribution in one millilitre of whole blood. This calculation was performed for each cell population according to the Equation (2).

Concentration of protein in one millilitre of whole blood.

2 $$\frac{Cell\,number\,of\,cell\,population\,in\,1\,mL\,of\,whole\,blood}{Number\,of\,cell\,assayed\,per\,mL}\times Concentration\,of\,analyte\,per\,mL$$

For cytokine analysis, statistical analysis of raw fluorescence responses was performed using ‘R’ version 3.4.1 (2017–06–30, R: A Language and Environment for Statistical Computing). Mixed-effects modelling were done using lmer^40^. For post-hoc analysis the significance of interactions terms were obtained using the Phia package^41^. Multiple test correction was done according to the false discovery method^42^. For analysis of the recombinant cytokine binding to RBCs, the following mixed-effects model, in R notation, was used: log2(FI) ~Fluid * Cytokine + Treatment * Cytokine + Treatment:Fluid + (1|ID) + (1|Treatment:Fluid:Plate). Where the log2 of fluorescence responses (Fl) was modelled using 3 fixed effects, (1) Fluid (2 levels, lysate and secretion), (2) Cytokine (either 27 or 21 levels), and (3) Treatment (2 levels, Control, BP) plus their interactions, together with two random effects defined as (1:ID), where ID represented subject identifier, and (1|Treatment:Fluid:plate) and where plate was a factor containing 2 levels. For analysis of the washed RBCs cytokine expression levels, the following mixed-effects model, in R notation, was used: log2(FI) ~Tissue* Cytokine + (1|ID). Where the log2 of the fluorescence responses (Fl) was modelled using 2 fixed effects plus their interaction plus one random effect. Tissue represented a subset from the set: Cytosol, Lysate, Sec. 1 W (RBC conditioned media following one wash), and Sec. 10 (RBC conditioned media following 10 washes) depending on the experiment under consideration. Experiment 1 contained only Cytosol and Lysate tissues and Experiment 2 contained only Sec. 1 W and Sec. 10 W. Cytokine contained identifiers for either 21-plex or 27-plex analytes. The term in brackets (1|ID) (random intercept) represented the random effect for the patient identifiers. The random effect accounted for patient-to-patient variability and for the non-independence in the data due to multiple samples and analyte readings per subject. The median of the dynamic range of the cytokines in the experimental samples was determined to be above the Bio-Plex kit blank and thus indicative of real values (Supplementary Figure S1).

### Data availability

All data generated or analysed during this study are included in this published article (and its Supplementary Information files).

## Electronic supplementary material

Supplementary data
