# Supplementary material for: Red blood cells are dynamic reservoirs of cytokines
Source: Sci Rep. 2018 Feb 15;8:3101. doi: 10.1038/s41598-018-21387-w (PMC5814557; doi:10.1038/s41598-018-21387-w)
Supplement: Supplementary file 1 — Supplementary data [file 41598_2018_21387_MOESM1_ESM.pdf]

Red blood cells are dynamic reservoirs of cytokines.

Authors: *Elisabeth Karsten[1,2,3]; Edmond Breen[4]; Benjamin R. Herbert[1,2,3]

Affiliations: [1]Translational Regenerative Medicine Laboratory, Kolling Institute, Royal North Shore Hospital, Sydney, Australia; [2]Northern Clinical School, Faculty of Medicine, The University of Sydney, Sydney, Australia; [3]Sangui Bio Pty Ltd, Sydney, Australia; [4]Bioinformatic Consulting, Sydney, Australia

Correspondence: Elisabeth Karsten, Sangui Bio Pty Ltd, PO Box 4054, Royal North Shore Hospital, Sydney, NSW 2065, Australia; e-mail: elisabeth@sanguibio.com

Supplementary Table S1. Percentage of protein recovery after recombinant protein spike into red blood cell lysates for cytokines as measured by fluorescence (n = 10, mean ± SD).

Cytokine	Protein recovery after spike*	
	%	Standard deviation
**Pro-inflammatory**		
IFN-α2	87.4	29.3
IFN-γ	122.9	6.2
IL-1α	102.7	6.4
IL-1β	101.9	2.7
IL-5	102.2	5.0
IL-9	91.1	9.6
IL-12(p70)	103.8	11.9
IL-15	59.8	12.8
IL-17	96.3	6.6
IL-18	95.4	4.3
MIF	100.6	4.7
TNF-α	97.2	4.0
TNF-β	100.8	4.1
TRAIL	106.0	4.2
**Anti-inflammatory**		
IL-1ra	106.4	3.2
IL-2ra	88.1	5.0
IL-4	95.9	4.0
IL-10	92.5	2.3
IL-13	94.7	12.1
**Cytokines with multiple functions**		
IL-2	82.4	13.3
IL-6	94.0	4.4
IL-12(p40)	140.5	41.5
LIF	98.7	4.6

Supplementary Table S2. Percentage of protein recovery after recombinant protein spike into red blood cell lysates for chemokines and growth factors as measured by fluorescence (*n* = 10, mean ± SD).

Cytokine	Protein recovery after spike*	
	%	Standard deviation
	Chemokines	
CTACK	90.6	17.7
Eotaxin-1	62.4	5.7
GRO-a	33.5	10.7
IL-8	72.3	8.6
IL-16	109.1	17.4
MCP-1	68.2	7.7
MCP-3	48.9	8.6
MIG	103.2	6.9
MIP-1α	88.1	3.4
MIP-1β	55.1	6.5
RANTES	79.0	20.0
SDF-1α	62.9	7.6
	Growth factors	
bFGF	111.2	11.9
G-CSF	89.8	18.6
GM-CSF	95.8	2.8
HGF	72.9	6.3
IL-3	120.4	6.1
IL-7	89.8	5.7
IP-10	93.1	4.3
M-CSF	94.6	4.7
β-NGF	93.5	6.1
PDGF-bb	91.4	2.6
SCF	113.9	4.2
SCGF-β	99.1	7.7
VEGF	113.2	5.2

Supplementary Table S3. Cytokines in red blood cell (RBC) conditioned media after 24 hours at 37 °C (200 million cells/mL) (n = 10, mean ± SD).

Cytokine	RBC cytokine release		
	**Concentration (pg/mL)**	**Standard deviation**	**No. of subjects**
	**Pro-inflammatory**		
IFN-α2	19.3	3.3	6
IFN-γ	3.7	2.2	4
IL-1α	6.7	3.8	10
IL-1β	18.0	18.8	10
IL-5	0.2	0.2	2
IL-9	5.7	5.6	5
IL-12(p70)	3.1	1.7	4
IL-15	12.3	5.2	9
IL-17	11.5	1.9	5
IL-18	4.8	3.1	10
MIF	1558.4	570.5	10
TNF-α	57.6	45.2	10
TNF-β	3.6	0.4	3
TRAIL	19.7	7.1	8
	**Anti-inflammatory**		
IL-1ra	19.2	15.0	3
IL-2ra	15.3	5.9	10
IL-4	7.5	12.5	8
IL-10	3.4	2.3	4
IL-13	0.4	0.2	5
	**Cytokines with multiple functions**		
IL-2	5.8	2.3	9
IL-6	5.9	3.4	8
IL-12(p40)	197.6	179.5	9
LIF	10.6	1.9	3

Supplementary Table S4. Chemokines and growth factors in red blood cell (RBC) conditioned media after 24 hours at 37 °C (200 million cells/mL) (*n* = 10, mean ± SD).

Cytokine	RBC cytokine release		
	**Concentration (pg/mL)**	**Standard deviation**	**No. of subjects**
**Chemokines**			
CTACK	59.9	55.2	5
Eotaxin-1	42.7	23.9	4
GRO-a	29.2	3.3	3
IL-8	657.1	788.2	10
IL-16	31.5	36.7	8
MCP-1	15.9	12.1	9
MCP-3	-	-	0
MIG	6.1	3.8	10
MIP-1α	2.9	2.4	9
MIP-1β	54.2	51.3	9
RANTES	235.8	179.6	10
SDF-1α	69.0	24.8	7
**Growth factors**			
bFGF	37.0	8.2	10
G-CSF	8.9	3.3	9
GM-CSF	97.5	38.1	9
HGF	41.5	17.9	7
IL-3	26.2	11.5	7
IL-7	0.2	-	1
IP-10	11.0	12.3	2
M-CSF	4.8	2.6	9
B-NGF	-	-	0
PDGF-bb	23.2	44.9	10
SCF	3.8	1.0	3
SCGF-β	-	-	0
VEGF	20.7	7.5	10

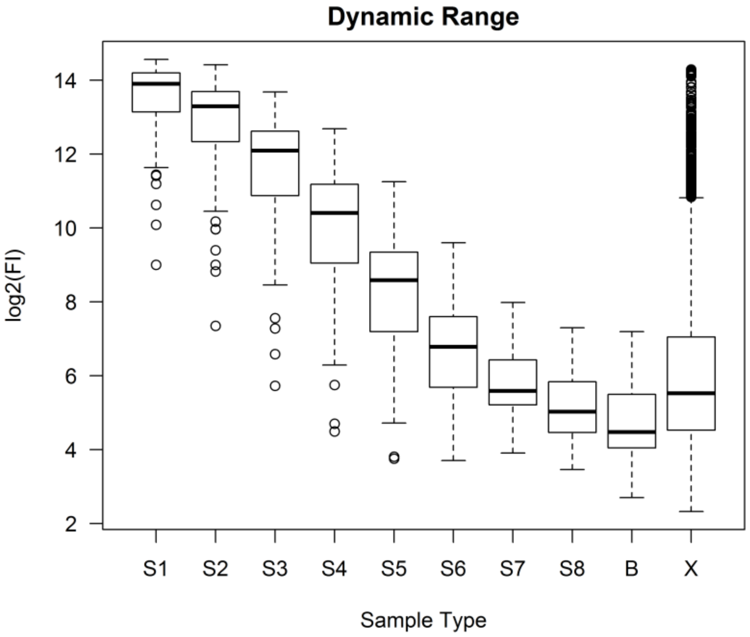

Supplementary Figure S1. Box plot of the dynamic range of all assayed cytokines in Bio-Plex kit standards (S1 – S8), Bio-Plex kit blank (B), or experimental samples (X) as measured by Bio-Plex and reported as fluorescence. Experimental samples include RBC lysates, cytosol, and RBC conditioned media samples. Data are presented as median with minimum and maximum values.
